# Successful Management of a Recurrent Supralevator Abscess: A Case Report

**DOI:** 10.1155/2012/871639

**Published:** 2012-05-07

**Authors:** S. Sanyal, F. Khan, Prashanth Ramachandra

**Affiliations:** Drexel University College of Medicine, Philadelphia, PA 19129, USA

## Abstract

Anorectal abscesses are commonly encountered in clinical surgical practice. These abscesses require surgical management. Supralevator abscesses are thought to originate either from an ischiorectal or intersphincteric abscess extension or from an intraperitoneal source. These abscesses are quite uncommon and present a difficult surgical problem. We present a case here of a 42-year-old female with a recurrent supralevator abscess requiring multiple surgical procedures for adequate drainage and care of her abscess.

## 1. Introduction

Abscesses may commonly occur in the Anorectal area. Anorectal abscesses seem to be most commonly found in males (1.76 : 1) in their third through fifth decades [1], with risk factors including foreign bodies, malignancy, trauma, tuberculosis, actinomycosis, leukemia, postoperative infection, inflammatory bowel disease, and simple skin infections [2]. Anorectal abscesses are classified according to their location. Most commonly, these occur in the perianal region (44.8%), followed by intermuscular (28%), and ischiorectal (12.8%). Supralevator abscesses are relatively rare, occurring in only 3.6% of Anorectal abscesses [2]. The primary event in abscess formation is infection of the anal glands located in the anal crypts along the dentate line. Afterwards, in a supralevator abscess, there is first involvement of the intersphincteric plane followed by upwards spread above the levator ani. However, supralevator abscesses are somewhat unique in that another potential source of the infection is from above, from a pelvic process such as diverticular disease or Crohn's disease [3]. Due to the abundant neural plexus tissue in the supralevator area, patients with these abscesses may have, in addition to anorectal pain and fever, presentations due to nervous involvement such as urinary retention or, rarely, sciatica [4]. Furthermore, patients may present with only abdominal or pelvic complaints without any anorectal complaints or findings. The treatment for a supralevator abscess is drainage. Antibiotics alone are inadequate and failure of timely drainage can result in significant morbidity. If untreated, a supralevator abscess can invade adjacent spaces and tissues, forming a necrotizing infection or an anal fistula. Such a fistula may also result from surgical incision and drainage of an abscess, although care is taken to keep the drainage site as close to the anal sphincter complex as possible, so that if a fistula were to form, its tract would be shorter [5].

## 2. Case

**Figure 1:**
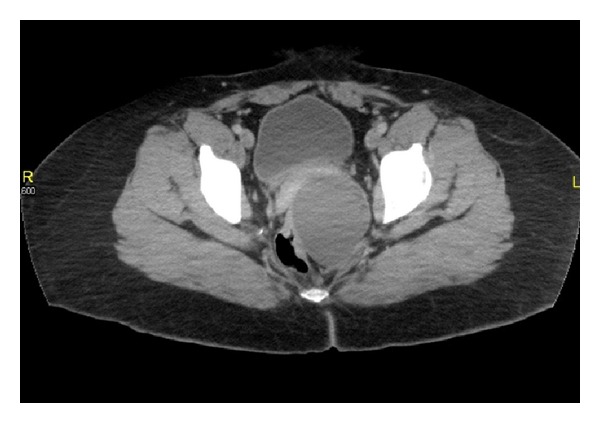
CT scan showing 7.5 × 6.4 × 6.6 cm abscess.

The patient was a 42-year-old female who initially presented to the ED with left buttock and left-sided back pain that was described as “12/10.” She had previously had a left gluteal abscess incised and drained at an outside hospital. She denied any history of fever. Her other past medical history included schizophrenia and prior Caesarian sections. She had a 6-pack-year smoking history. On physical examination, the patient was afebrile with stable vitals. She was awake and alert. Her abdomen was soft, obese, and nontender without masses. Rectal examination showed good sphincter tone without any purulent drainage. A boggy swelling was palpable in the left side of the rectum at the 3 or 4 o'clock position. The rest of the physical exam was benign. A CAT (computerized axial tomography) scan was performed, showing a large right-sided abscess (Figure 1)

The patient was taken to the operating room for examination under anesthesia. Digital rectal exam showed an induration and boggy swelling 10 cm from the anal verge and at the 3 o'clock position. The examination was complemented with anoscopy, which revealed vague bulge into the lumen corresponding to the digital palpation site. Due to the difficult location of the abscess, surgical drainage was not feasible transrectally and the abscess was drained percutaneously under fluoroscopy by Interventional radiology.

**Figure 2:**
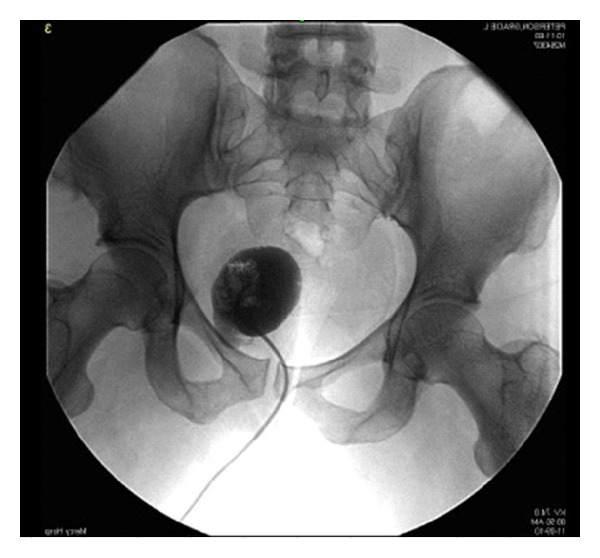
Initial abscessogram for transgluteal drainage.

**Figure 3:**
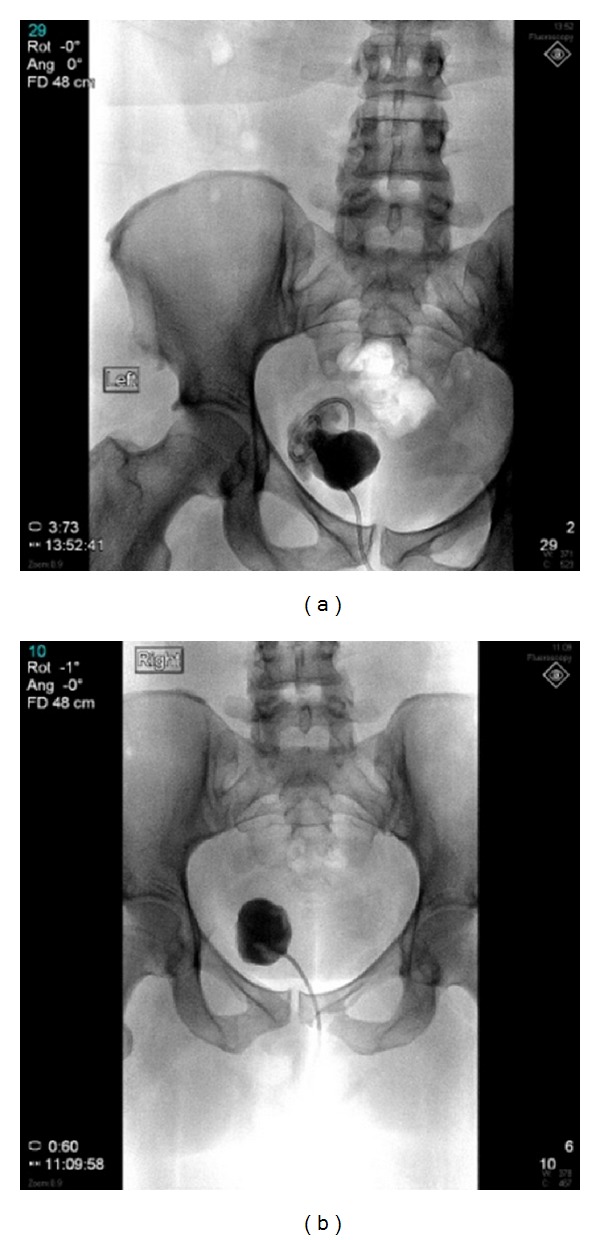
Abscessograms from tube checks showing a visibly smaller abscess.

Multiple pictures were taken under fluoroscopy to characterize the abscess, shown in Figures 2 and 3. The drainage catheter was left creating a fistula between the abscess and the skin. The drainage catheter had several dislodgements. Two tube-checks and adjustments were done by interventional radiology. 

However, two months after the initial procedure, the patient was again admitted with the similar symptoms. The patient was taken for surgical drainage through the previous transgluteal catheter insertion site, whereupon mucopurulent material was drained. The abscess was packed and the patient was once again discharged on trimethoprim/sulfamethoxazole and ciprofloxacin. One month after this, the patient was again admitted complaining of pain in her abdomen, groin, and vagina and drainage of purulent material from the left buttock at the surgical incision site. The patient was taken to the OR and an incision was made through her previous incision scar in the left buttock about 6 cm from the anal opening. Bimanual exam revealed a large boggy swelling. The abscess was dissected out and then opened. The serous and purulent fluid contents were drained and a size 28 Malecot and a 22 French Foley were both left in and sutured to the skin. The patient tolerated the procedure well and was sent home with daily wound and drain care as well as a 10 day course of ciprofloxacin and trimethoprim/sulfamethoxazole. She returned to the outpatient office for followup and had complete recovery of her abscess.

## 3. Discussion

Supralevator abscess presents within the potential pelvirectal or supralevator space, which lies between the pelvic floor and the levator ani muscles [6]. It is imperative that adequate drainage be performed, even if this necessitates aggressive surgical intervention. The intensive surgical course of this patient highlights the difficult nature of this disease. Although the supralevator abscess is rare [7], it requires much more aggressive treatment than other locations of Anorectal abscesses. Drainage can be attempted through many approaches. It is difficult to access supralevator abscess rectally and drain it adequately. If drained through levator ani muscle through ischiorectal fossa a complex fistula can result and recurrences are common [7]. This leaves percutaneous drainage or open transabdominal drainage. While percutaneous drainage has several advantages and is minimally invasive, it can often lead to inadequate results, as in this case. Definitive treatment can be guaranteed through an open transabdominal approach, allowing for good visualization of the abscess and ensuring adequate drainage.

## 4. Conclusion

The diagnosis of supralevator abscess is not easily made clinically due to anatomical location and requires imaging. It is important to recognize the possibility of a supralevator abscess whenever a patient presents with rectal, pelvic, or back pain and signs of infective process. This case study demonstrates that inadequate management of a supralevator abscess upon first presentation can lead to higher morbidity and require further interventions. This is especially true with patients who may be noncompliant with follow-up visits. It is important in these cases to make sure that the abscess is properly managed upon first presentation. Percutaneous drainage of this difficult abscess subtype might be considered as a viable option, but may need further exploration as compared to open drainage of the supralevator abscess. The open drainage has an inherent higher complication rate particularly in patients with multiple comorbidities.
